# Topoisomerase II Inhibitors Can Enhance Baculovirus-Mediated Gene Expression in Mammalian Cells through the DNA Damage Response

**DOI:** 10.3390/ijms17060931

**Published:** 2016-06-14

**Authors:** Ming-Kun Liu, Jhe-Jhih Lin, Chung-Yung Chen, Szu-Cheng Kuo, Yu-Ming Wang, Hong-Lin Chan, Tzong Yuan Wu

**Affiliations:** 1 Institute of Bioinformatics and Structural Biology, National Tsing Hua University, Hsinchu 300, Taiwan; lmk33@hotmail.com (M.-K.L.); blackeye514@hotmail.com (J.-J.L.); hlchan@life.nthu.edu.tw (H.-L.C.); 2 Department of Bioscience Technology, Chung Yuan Christian University, Chungli 320, Taiwan; cychen@cycu.edu.tw; 3 Institute of Preventive Medicine, National Defense Medical Center, Taipei 237, Taiwan; szucheng@mail.ndmctsgh.edu.tw (S.-C.K.); yuming0724@gmail.com (Y.-M.W.); 4 Department of Medical Research, China Medical University Hospital, China Medical University, Taichung 404, Taiwan

**Keywords:** baculovirus, BacMam, DNA damage response, topoisomerase, p53

## Abstract

BacMam is an insect-derived recombinant baculovirus that can deliver genes into mammalian cells. BacMam vectors carrying target genes are able to enter a variety of cell lines by endocytosis, but the level of expression of the transgene depends on the cell line and the state of the transduced cells. In this study, we demonstrated that the DNA damage response (DDR) could act as an alternative pathway to boost the transgene(s) expression by BacMam and be comparable to the inhibitors of histone deacetylase. Topoisomerase II (Top II) inhibitor-induced DDR can enhance the CMV-IE/enhancer mediated gene expression up to 12-fold in BacMam-transduced U-2OS cells. The combination of a Top II inhibitor, VM-26, can also augment the killing efficiency of a p53-expressing BacMam vector in U-2OS osteosarcoma cells. These results open a new avenue to facilitate the application of BacMam for gene delivery and therapy.

## 1. Introduction

Baculoviruses are enveloped, double-stranded DNA viruses belonging to the *Baculoviridae* family and can infect over 600 insect species [1]. Among the numerous baculovirus species, *Autographa californica* multiple nucleopolyhedrovirus (AcMNPV) is the prototype baculovirus for basic virology studies and biotechnology applications [2]. The genome of AcMNPV (approximately 134 kb) is packaged into a rod-shaped nucleocapsid, typically 40–50 nm in diameter and 200–400 nm in length [3,4]. The first successful genetic recombinant AcMNPV carrying the human β-interferon gene was generated using a homologous recombinant approach in fall armyworm-derived Sf21 cells in the early 1980s [5]. This study demonstrated that the functional recombinant β-interferon protein can be produced by the recombinant baculovirus, thus launching a new tool for recombinant protein production and making AcMNPV one of the most popular genetic vehicles. Since then, the insect cell-based baculovirus expression vector system (BEVS) has been routinely used in both basic research and industrial laboratories to produce diverse types of recombinant proteins for research, medical, agricultural, and veterinary applications [2,6]. Typical examples, such as the cervical cancer vaccine Cervarix^®^ developed by GlaxoSmithKline [7] and the flu vaccine FlublØck^®^ developed by Protein Sciences Corporation [8], are both BEVS-derived products. In addition to being a successful recombinant protein expression system, by engineering the genome of AcMNPV with promoters that are derived from mammalian cells or viruses, the recombinant AcMNPV can also mediate gene expression in mammalian cells and has emerged as a valuable genetic delivery vehicle [9]. In 1995, Hofmann *et al.* reported that a recombinant AcMNPV was able to mediate *E. coli* β-galactosidase gene (*lacZ*) or firefly luciferase gene expression in hepatocytes provided that both the reporter genes’ expression was driven by the cytomegalovirus-derived immediate early promoter (CMV-IE)/enhancer [10]. Subsequently, in 1996, Boyce and Bucher also depicted efficient baculovirus-mediated expression of the *lac*Z gene under the control of the Rous Sarcoma Virus (RSV) promoter in the hepatoma cell line HepG2 and primary rat hepatocytes [11]. These two pioneering studies suggested that only liver-derived cells can be transduced efficiently by recombinant baculoviruses and implied that the recombinant baculoviruses can act as a liver-specific gene delivery tool [10,11]. Based on these interesting observations, Shoji *et al.* then demonstrated that recombinant baculovirus can mediate gene expression efficiently in non-hepatic cells, such as HeLa and COS-7 cells, by a chimeric CAG promoter consisting of a CMV immediate-early enhancer, chicken β-actin promoter, and rabbit β-globin polyadenylation signal [12]. Since then, the cell lines and primary cells efficiently transduced by recombinant baculovirus have significantly expanded and even include fish cells [13,14]. Thus, this safe, easily manipulated, and scaled-up recombinant AcMNPV has been explored in gene delivery, the surface display of eukaryotic proteins, and cell-based assays for drug development and cancer therapy both *in vitro* and *in vivo* [15,16]. Recently, combining the sodium iodide symporter (NIS) reporter gene with image technology, the AcMNPV vector can also be employed to monitor the cell fate of human stem cells *in vivo*. These recombinant AcMNPVs that were adapted for multiple purposes for use in mammalian cells are called BacMam [17].

To act as a gene delivery vector, biosafety is a top priority. In this regard, BacMam has a profound biosafety profile compared with other gene delivery vectors based on mammalian viruses [6] and is recognized as a risk group I agent. There is no evidence that baculovirus causes diseases in vertebrate organisms with productive or latent viral infection in vertebrate cells [18]. This safety insurance promotes the study and development of versatile BacMam vectors. To prolong the transgene expression, the sleeping beauty expression technique has been successfully used in tissue engineering by BacMam [19,20]. Recently, the transcription activator-like effector nucleases (TALEN) module was introduced into BacMam and offers the potential to manipulate genes precisely in stem cells [21].

When BacMam is applied as a gene delivery vector, the transduction rate is of great concern and is critical for successful applications. The transduction rate of BacMam depends on (1) the efficiency of entry of the viruses into the cells and transportation into the nucleus and (2) the level of expression of the transgene in the transduced cells [22]. Previous studies have demonstrated that BacMam vectors can enter every tested cell line by endocytosis [23,24]. However, the expression level of the transgene depends on the cell line and the state of the transduced cells. Based on this observation, it is critical to find ways to manipulate the cell state(s) to successfully apply BacMam as a gene therapy or delivery vector. The pan-histone deacetylase inhibitor (HDACi) sodium butyrate (NaBt) can affect the epigenetic state of cells and enhance gene expression in eukaryotic cells [25]. For example, transgene expression under the control of the CMV-IE/enhancer of an engineered BacMam vector can be enhanced more than 10-fold by NaBt in HeLa cells [25]. Thus, it is implied that the BacMam vector delivers the CMV-IE/enhancer-controlled cassette into the nucleus, where it underwent silencing directly or indirectly by histone deacetylation, which can be alleviated by the inhibition of histone deacetylase (HDAC) through HDACis, such as NaBt [18]. In the present study, we provide evidence that the DNA damage response (DDR) could act as an alternative way to boost the transgene(s) expression by BacMam. The topoisomerase II (Top II) inhibitors that induce DDR can augment CMV-IE/enhancer-mediated gene expression in BacMam-transduced human osteosarcoma U-2OS cells or human hepatocellular carcinoma (HepG2) cells. The combination of the Top II inhibitor VM-26 to increase the killing efficiency of the p53-expressing BacMam vector was also demonstrated.

## 2. Results

### 2.1. VM-26 Can Enhance Baculovirus-Transduced Gene Expression in U-2OS Cells

**Figure 1:**
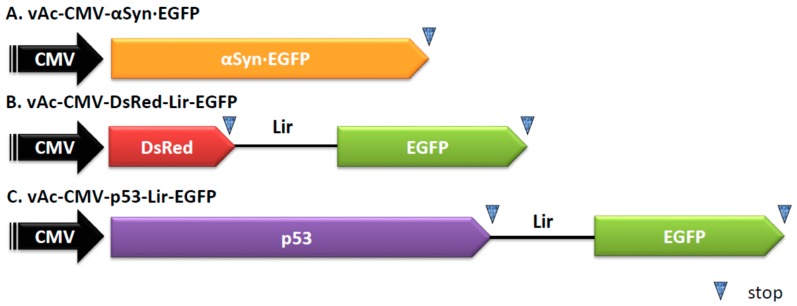
BacMam vectors used in this study. (**A**) vAc-CMV-αSyn·EGFP is the baculovirus that contains the human cytomegalovirus immediate early promoter (CMV) to control the expression of the α-synuclein-EGFP fusion protein (αSyn·EGFP); (**B**) vAc-CMV-DsRed-Lir-EGFP is a bi-cistronic baculovirus that uses the CMV promoter to control the expression of bi-cistronic mRNA DsRed-Lir-EGFP. DsRed, the red fluorescent protein; Lir, a chimeric IRES; EGFP, enhanced green fluorescent protein; (**C**) vAc-CMV-p53-Lir-EGFP is a bi-cistronic baculovirus that uses the CMV promoter to drive the expression of bi-cistronic mRNA p53-Lir-EGFP. p53 is the tumor suppressor protein.

**Figure 2:**
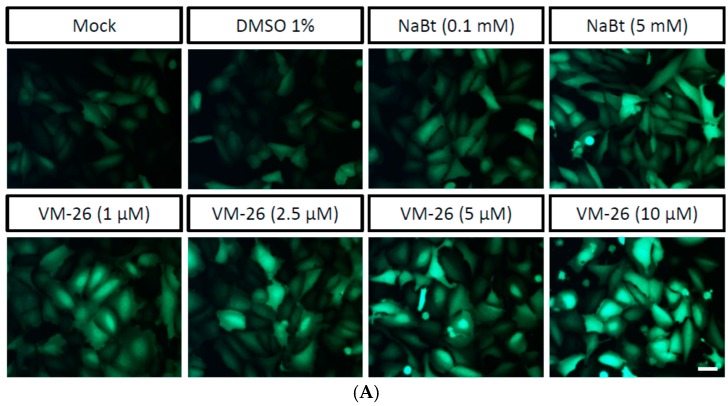

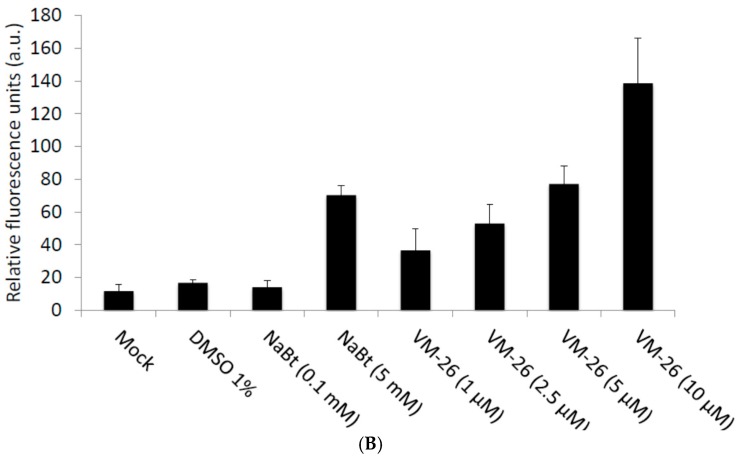
VM-26 can enhance BacMam transduction of U-2OS cells. (**A**) Representative images of U-2OS cells (5 × 10^4^ cells seeded in a 24-well plate) transduced with vAc-CMV-αSyn·EGFP (moi = 100) and then concomitantly treated with NaBt or VM-26 at the indicated concentration for 24 h. A mock treatment or 1% DMSO was used as a control. Pictures were taken 24 h after transduction under a FITC channel with a 450/490-nm filter set using an exposure time of 1 s; the scale bar is 50 μm; (**B**) Spectrofluorometric measurement of EGFP fluorescence from U-2OS cells transduced with vCMV-αSyn·EGFP after 24 h and treated with NaBt or VM-26 as indicated. EGFP fluorescence emission was excited at 488 nm and monitored at 507 nm. All of the data are presented as the mean ± SD of three independent experiments.

The DNA damage response (DDR) can be adapted or employed by many DNA viruses to facilitate virus infection [26,27]. For example, the mammalian herpesviruses [28] and insect baculoviruses [29] can use the host DDR for efficient viral gene expression and viral DNA replication. Based on these observations, we tested whether the induction of DNA double-strand breaks by VM-26, a topoisomerase II inhibitor and a DDR inducer, can enhance the gene expression mediated by baculovirus in transduced mammalian cells. We first found that when the U-2OS cells were transduced with vAcCMV-αSyn·EGFP (Figure 1A) and then treated with VM-26, the expression of green fluorescence was enhanced under a fluorescent microscope observation (Figure 2A). Then, using a spectrofluorometer to quantify the expression of the EGFP protein, we also revealed a dose-dependent enhancement by VM-26 (Figure 2B). Interestingly, the fold change in EGFP intensity upon 5 μM VM-26 treatment was similar to that of 5 mM NaBt-treated cells (Figure 2B). This result suggests the DDR can enhance the gene expression in U-2OS cells when transduced with BacMam.

### 2.2. Top II but Not Top I Inhibitors Enhance Baculovirus-Delivered Gene Expression in U-2OS Cells

**Figure 3:**
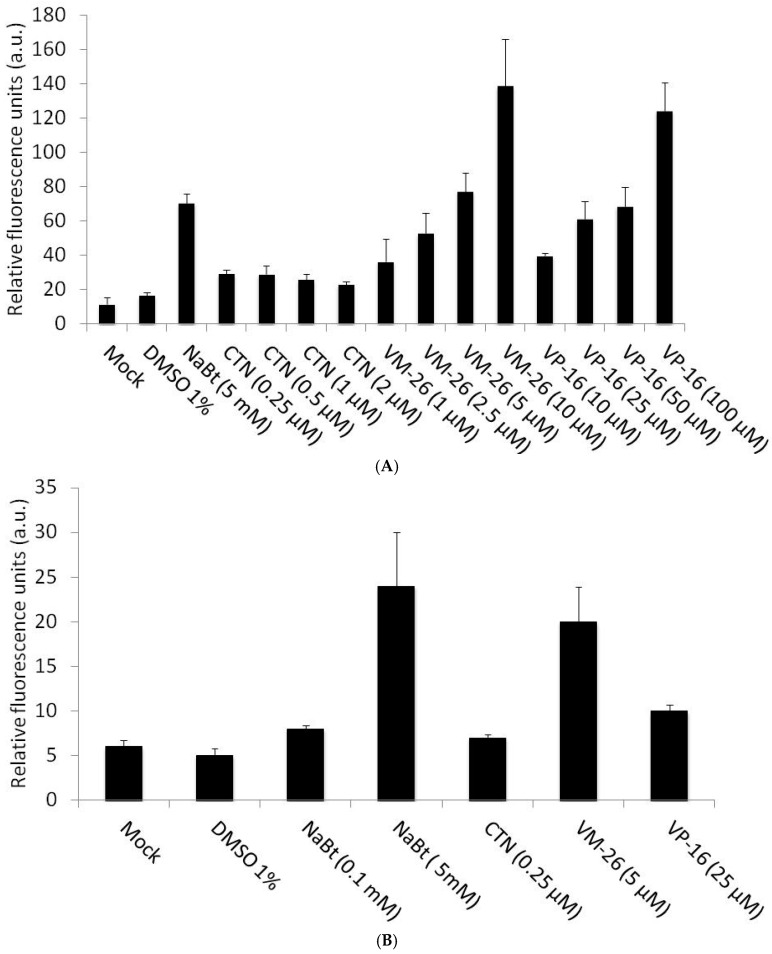
Top II inhibitors enhance BacMam-mediated gene expression in U-2OS cells more than a Top I inhibitor, camptothecin (CTN). (**A**) Spectrofluorometric measurement of EGFP fluorescence from U-2OS cell lysates (5 × 10^4^ cells seeded in a 24-well plate) transduced with vAc-CMV-αSyn·EGFP (moi = 100) and in the presence of CTN (0.25–2 µM), a Top I inhibitor, or Top II inhibitors, VM-26 (1–10 µM) and VP16 (10–100 µM) as indicated for 24 h; (**B**) Spectrofluorometric measurement of EGFP fluorescence from HepG2 cell lysates (5 × 10^4^ cells seeded in a 24-well plate) transduced with vAc-CMV-αSyn·EGFP (moi = 100) after 24 h in the presence of CTN (0.25 µM), VM-26 (5 µM) and VP16 (25 µM). EGFP fluorescence emission was excited at 488 nm and monitored at 507 nm. Mock treatments, 1% DMSO or NaBt were used as controls. All of the data are presented as the mean ± SD of three independent experiments.

DDR can be induced by versatile topoisomerase inhibitors but with different mechanisms. For example, VM-26 is a Top II inhibitor and can cause cellular DNA double-strand breaks to induce DDR, while camptothecin (CTN), a Top I inhibitor, can induce the DDR but through a cellular DNA single-strand break [30,31]. To elucidate whether the DDR induced by Top I inhibitors, Top II inhibitors, or both can facilitate the gene expression delivered by baculoviruses, Top I inhibitor CTN and Top II inhibitors VM-26 and VP-16 were tested. We found that the Top I inhibitor CTN (0.25–2 μM) did not enhance baculovirus-mediated gene expression in U-2OS cells effectively and did not follow the dose response (Figure 3A). In contrast, the enhanced EGFP expression in the vAcCMV-αSyn·EGFP-transduced U-2OS cells was observed in the presence of VM-26 (1–10 μM) and VP-16 (10–100 μM) (Figure 3A). It is interesting to note that 10 μM VM-26 can enhance BacMam-mediated gene expression more potently than 10 μM VP-16; thus, we used VM-26 in the subsequent experiments. To test whether these observations were also true in other cell lines, we used CTN, VM-26, and VP-16 to treat vAcCMV-αSyn·EGFP-transduced hepatocellular carcinoma HepG2 cells. Figure 3B showed that both VM-26 (5 μM) and VP-16 (25 μM), but not CTN (0.25 μM), can enhance EGFP expression in the vAcCMV-αSyn·EGFP-transduced HepG2 cells. Thus, the Top II inhibitors rather than Top I inhibitors can enhance the baculovirus-transduced gene expression in U-2OS or HepG2 cells.

### 2.3. VM-26 Enhanced Baculovirus Gene Expression in U-2OS Cells through the DDR but Does Not Alter the Acetylation of Histone Proteins

**Figure 4:**
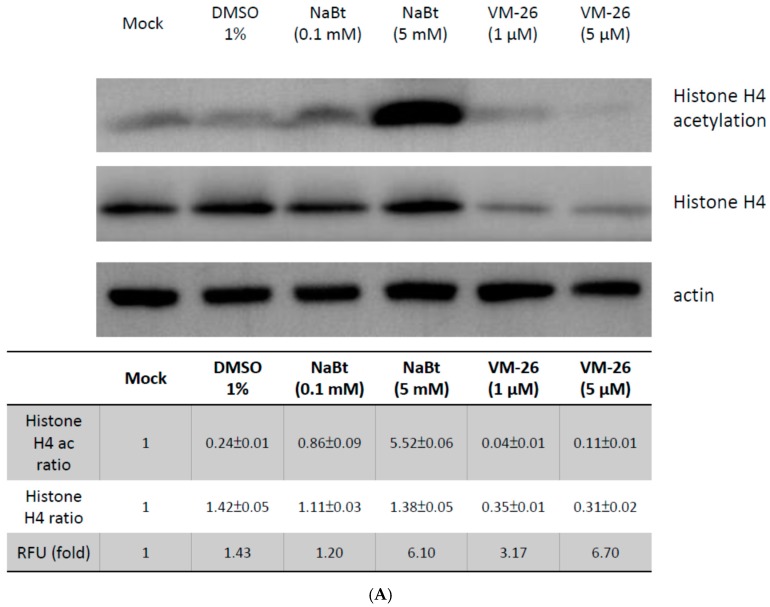

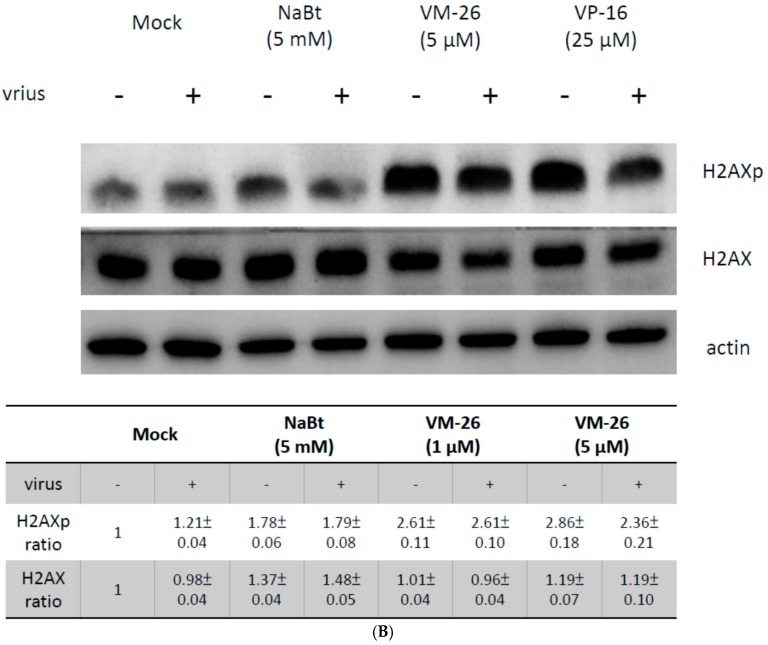
VM-26 can enhance BacMam-mediated gene expression in U-2OS cells by DDR but does not change the acetylation state of histone proteins. (**A**) Western blot analysis of acetylated-histone H4 in U-2OS cells (5 × 10^4^ cells seeded in a 24-well plate) treated with NaBt (0.1 or 5 mM) or VM-26 (1 or 5 µM) by anti-histone H4 acetylation antibody or anti-histone H4 antibody, respectively. Signal intensity of acetylated-histone H4 (histone H4 ac ratio) and non-modified histone H4 (histone H4 ratio) in the Western blot were quantified by Image J software and normalized to the intensity of actin present in the lower panel. RFU (fold) indicated the enhancement of EGFP expression as measured by spectrofluorometer in the vAc-CMV-αSyn·EGFP transduced U-2OS cells under the indicated treatment; (**B**) The DDR were detected by the presence of H2AXp by immunoblot probed with an anti-H2AXp antibody. U-2OS cells (5 × 10^4^ cells seeded in a 24-well plate) treated with sodium butyrate or VM-26 were transduced with vAc-CMV-αSyn·EGFP (moi = 100, +) or not transduced (−). After 24 h of transduction, the U-2OS cells were lysed and immunoblots were performed with anti-H2AXp antibody or anti-H2AX antibody, respectively. The fold of vAc-CMV-αSyn·EGFP (moi = 100) mediated αSyn·EGFP gene expression in U-2OS cells in the presence of NaBt or VM-26 was determined by spectrofluorometer as mentioned in the Materials and Methods section and is presented as the mean ± SD of three independent experiments. Actin was used as a loading control. Quantification of the H2AXp and H2AX band intensities relative to the expression of actin was presented in the lower panel. All of the data are presented as the mean ± SD of three independent experiments.

Previous studies have indicated that HDAC inhibitors such as NaBt or trichostatin A (TSA) can stabilize the acetylation state of histone proteins and then increase baculovirus-mediated exogenous gene expression in mammalian cells [25,32]. To dissect whether enhancement of baculovirus-mediated gene expression by VM-26 in U-2OS cells is due to an HDAC inhibitor-like increase in the acetylation of histone proteins, the acetylation of histone H4 protein of VM-26-treated U-2OS cells was analyzed with a Western blot probed with an anti-histone H4 acetylation antibody. Figure 4A showed that 5 mM NaBt significantly increased the acetylation of the histone H4 protein when compared with 1% DMSO. However, VM-26 (10 μM) could not increase the acetylation of histone H4 protein (Figure 4A, upper panel). In contrast, Figure 4B shows that VM-26 and VP-16 induced DDR as revealed by the phosphorylation of H2AX proteins (H2AXp, an indicator of DDR), but NaBt did not change the phosphorylation state of H2AX proteins. Our data suggest that it was possible that the DDR can also enhance baculovirus-mediated gene expression in mammalian cells, independent of stabilizing the acetylation state of histone proteins.

As the DDR is induced by topoisomerase inhibitors, such as VP-16, topoisomerase II would be degraded through the 26S proteasome [33]. This fact may imply that the 26S proteasome might also degrade the histone proteins and change the epigenetic states of the DDR-induced cells. Therefore, the efficiency of gene expression mediated by baculoviruses has improved. Thus, we analyzed whether the histone H4 protein was also degraded after the DDR was induced by VM-26 in U-2OS cells. As expected, the level of histone H4 protein in the cells was decreased after treatment with VM-26 but was not changed when cells were treated with NaBt (Figure 4A, lower panel). These results suggested that the reduced level of histone proteins by DDR affects the gene expression in mammalian cells mediated by BacMam.

### 2.4. Combined VM-26 with Baculovirus Mediated p53 Gene Therapy

**Figure 5:**
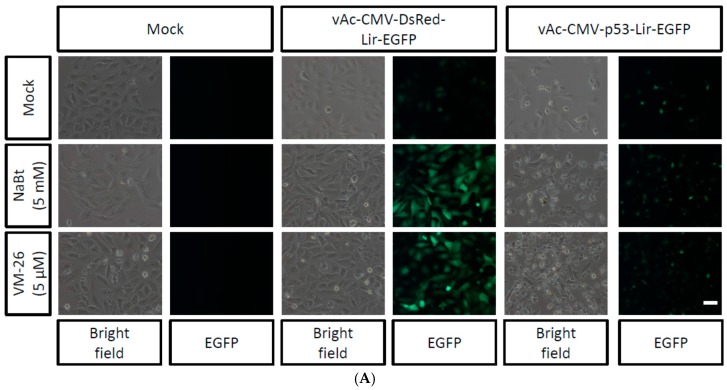

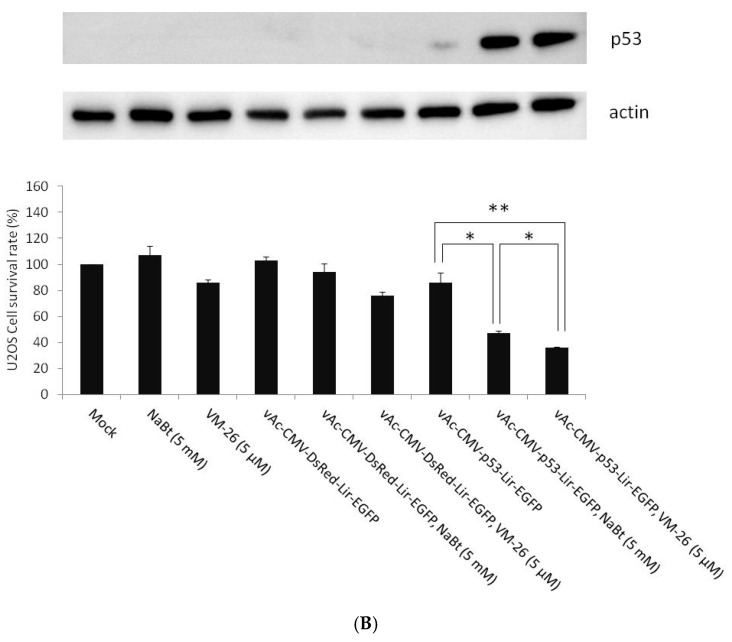
VM-26 enhances the BacMam-mediated p53 gene therapy effect in U-2OS cells. (**A**) Representative images of mock transduction, vAc-CMV-DsRed-Lir-EGFP (moi = 100), or vAc-CMV-p53-Lir-EGFP (moi = 100) transduced U-2OS cells (5 × 10^4^ cells seeded in a 24-well plate) that were concomitantly treatment with NaBt or VM-26 for 24 h. Pictures were taken 24 h after transduction under bright field or a FITC channel with a 450/490-nm filter set using an exposure time of 1 s; scale bar is 50 μm; (**B**) The cell viability of (**A**) was determined by MTT assay as described in the Materials and Methods. The upper panel displays the Western blot analysis of p53 proteins in the cell lysates of (**A**) probed with an anti-p53 antibody. Actin was used as a loading control. * *p* < 0.05 when compared with the corresponding control groups; ** *p* < 0.005 when compared with the corresponding control groups.

Baculovirus-delivered p53 gene expression has been explored for cancer gene therapy [34]. Previous studies revealed that combining HDAC inhibitors, *i.e.*, NaBt or TSA, with baculovirus-mediated p53 gene expression can dramatically enhance the efficacy of cancer cell killing [18]. Interestingly, both Top I and Top II inhibitors have been used in cancer chemotherapy regimens [31]. Thus, it is interesting to test whether the combination of VM-26 with baculovirus-mediated p53 gene expression can also enhance the efficacy of cancer cell killing. To test this hypothesis, the bi-cistronic baculovirus vAc-CMV-p53-Lir-EGFP was generated (Figure 1C). vAc-CMV-p53-Lir-EGFP contains a chimeric IRES, Lir, which can mediate gene expression in insect cells and mammalian cells. The Lir IRES was generated through the fusion of 110 nts of the RhPV IRES [35] and 100 nts of the truncated EV 71 IRES [36]. vAc-CMV-p53-Lir-EGFP can be isolated, purified, and quantified in insect cells through the expression of EGFP under a fluorescent microscope [36]. The p53 gene of vAc-CMV-p53-Lir-EGFP was replaced with a DsRed gene and generated a recombinant baculovirus vAc-CMV-DsRed-Lir-EGFP (Figure 1B) as a control. The U-2OS cells were transduced with vAc-CMV-DsRed-Lir-EGFP and vAc-CMV-p53-Lir-EGFP and further incubated in the presence of NaBt (5 mM) or VM-26 (5 μM) for 24 h. The vAc-CMV-DsRed-Lir-EGFP-transduced U-2OS cells emitted green fluorescence, and the intensity was significantly enhanced when NaBt or VM-26 was present (Figure 5A). The vAc-CMV-p53-Lir-EGFP-transduced U-2OS cells also emitted green fluorescence; however, the cellular morphology was changed, the cells appeared to shrink, and membrane blebbing was observed (Figure 5A). An MTT assay was used to quantify the cellular survivable rate after the U-2OS cells were treated with NaBt or VM-26 alone or when the cells were also transduced with vAc-CMV-DsRed-Lir-EGFP or vAc-CMV-p53-Lir-EGFP. NaBt did not reduce the survival rate of U-2OS under the concentration tested, while VM-26 caused approximately 14% cell death (Figure 5B). The vAc-CMV-p53-Lir-EGFP-transduced U-2OS cells exhibited a minor toxicity of approximately 14% cell death, whereas vAc-CMV-DsRed-Lir-EGFP did not. (Figure 5B). After combining the vAc-CMV-DsRed-Lir-EGFP with NaBt or VM-26, the survival rate of U-2OS cells was only slightly decreased; approximately 6% and 24% cell death was observed relative to the control (Figure 5B), although NaBt or VM-26 can enhance baculovirus-mediated exogene expression. Interestingly, after combining vAc-CMV-p53-Lir-EGFP with NaBt or VM-26, the U-2OS cells did exhibit significantly increased death, as only 47% and 36% of the U-2OS cells survived (Figure 5B). Consistent with the MTT assay results, the level of p53 protein expressed in U-2OS was also enhanced by VM-26 and by NaBt (Figure 5B). These results suggested that VM26, a chemotherapy agent, can enhance cancer toxic gene expression, such as p53, mediated by the BacMam vector.

## 3. Discussion

BacMam is recognized as a potential safe and efficient viral vector for gene therapy. The successful use of the insect-derived baculovirus vector for glioma [18], prostate cancer [37,38] and hepatoma [39,40] has been reported. The use of BacMam for tissue engineering has also been investigated in bone or stem cell modification [16,41]. This study demonstrated that Top II inhibitors, such as VM26 or VP-16, could enhance baculovirus-mediated gene expression in U-2OS cells. To our knowledge, this is the first study to report that chemicals other than HDACis can enhance baculovirus-mediated exogene expression in mammalian cells. Using VM-26, which also acts as a potent cancer chemotherapy agent, we also illustrated that combining VM-26 with p53-expressing BacMam could significantly increase the killing efficacy of U-2OS cells.

It is well established that baculoviruses can enter most mammalian cell lines and be subjected to epigenetic regulation that determines the level of expression of the transgenes [25]. HDACis, such as NaBt or TSA, can inhibit HDAC activity and then maintain the hyperacetylation state of the histones. Thus, they are usually combined with the BacMam vectors to enhance transgene expression. For example, 5 mM NaBt significantly increased the acetylated histone H4 protein (Figure 4A). However, the VM-26 did not alter the acetylated histone H4 protein (Figure 4A), but the level of expression of the transgene was enhanced (Figure 2 and Figure 5). This result implied that the DDR is another way to enhance BacMam-mediated transgene expression levels in mammalian cells.

Viruses have been “selected” to manipulate cellular processes, such as apoptosis, autophagy, or DDR, to facilitate viral replication and reproduction [27]. Herpes simples virus 1 (HSV-1) infection of dividing cells induces the ATM (ataxia telangiectasia mutated) pathway of DDR and nullification of components of this pathway reduces the infection of HSV-1 [42]. Thus, DDR is a cellular process that could be “hijacked” or “adapted” in the lytic infection cycle of HSV-1 to boost viral gene expression [42,43]. It is interesting to note that the DDR induced by Top II inhibitors, such as VM-26 or VP-16, can facilitate HSV-1 viral DNA replication and gene expression [44]. The viral genome of baculovirus AcMNPV is large, approximately 134 kbp, similar in size to HSV-1. Considering the genome size similarity between HSV-1 and AcMNPV, we proposed that when AcMNPV was transduced into U-2OS, the AcMNPV genome would act like the HSV-1 genome and the transgene expression would be boosted when DDR was induced by VM-26. However, it was possible that the DDR would act directly on the CMV promoter. Indeed, there is a report indicating that the activity of the CMV promoter can be enhanced approximately two-fold when VM-26 was added after plasmid transfection [44]. However, as revealed in Figure 2 or Figure 5, VM-26 could activate the CMV promoter in the AcMNPV genome up to more than 10-fold. Thus, there are some unknown mechanisms of VM-26 activation of the transgene expression level in the BacMam-transduced mammalian cells.

Recently, the importance of DDR has also been documented in baculovirus-infected insect host Sf21 cells. Mitchell *et al.* reported that the baculovirus conserved replication factor late expression factors 7 (LEF-7) modifies the insect host DDR and enhances virus multiplication. Interestingly, LEF-7 suppresses the DDR-induced accumulation of phosphorylated histone variant H2AX (γ-H2AX) [29]. This action is different from most DNA viruses that activate the host DDR and also trigger γ-H2AX accumulation. However, as shown in Figure 4B, the VM-26- and VP-16-induced DDR, as revealed by the phosphorylation of H2AX proteins, was not suppressed in the vAc-CMV-αSyn·EGFP transduced U-2OS cells, even though LEF-7 may be expressed in U-2OS cells. Combining these observations, we propose that DNA viruses, such as HSV-1 or baculovirus, require DDR for viral gene expression or the transgene expression under the control of CMV promoter for their genome to enter mammalian cells and then be delivered into the nucleus. This speculation is consistent with the following observations: (1) HSV-1 infected epithelial cells can induce DDR and promote viral gene expression to undergo lytic infection. In contrast, HSV-1-infected neuronal cells will exhibit latent infection because DDR is not induced; (2) The HSV-1 latent infection in neuronal cells can be reactivated; viral genes can be expressed again, after the DDR was evoked by stress, such as heat shock, irradiation, or chemicals.

Based on these observations mentioned above, we hypothesized that when baculoviruses are transduced into mammalian cells, such as U-2OS or HepG2, they will exhibit like a latent HSV-1 infection and the expression of transgene(s) will be inhibited. This “latent” state of the baculovirus genome may behave like heterochromatin, and the level of transgene expression is low. Two approaches can be used to relieve this expression hurdle. The first is through epigenetic regulation by HDACis to change the heterochromatin into euchromatin and to increase transgene expression. In the second approach, like the reactivation process of latent HSV-1, the expression of the transgene(s) could also be enhanced when the DDR pathway is induced.

An important application of this finding is that the DDR activation chemical reagents, such as Top II inhibitors, are recognized as potent chemotherapy agents [31], although some severe adverse events of Top II inhibitors, such as treatment-related acute myeloid leukemia (t-AML), have been reported [45]. Thus, like the combination of baculovirus-mediated cancer gene therapy with HDACis, it is possible to combine the baculovirus-mediated cancer gene therapy with the Top II inhibitors. However, it is interesting to note that the overexpression of p53 was critical to mediate the cell killing efficiency. As shown in Figure 5B, minor p53 proteins were barely detectable when U-2OS cells were transduced with vAc-CMV-p53-Lir-EGFP in the absence of NaBt or VM-26. The low expression of p53 also resulted in a low death rate of the vAc-CMV-p53-Lir-EGFP-transduced cells. Similarly, when U-2OS cells were transduced with vAc-CMV-DsRed-Lir-EGFP, even in the presence of NaBt or VM-26, the cell killing rates were also low as measured via the MTT assay. We found that only when p53 was overexpressed (as shown in the Western blot of Figure 5B) in the vAc-CMV-p53-Lir-EGFP, in the presence of NaBt or VM-26, transduced U-2OS cells and the cell killing efficiency were enhanced approximately two-fold. The U-2OS osteosarcoma cells are a p53 wild type cell line; therefore, it may be interesting to test vAc-CMV-p53-Lir-EGFP-mediated p53 gene expression in osteosarcoma cell lines expressing mutated p53 (MG-63) or p53 null (SAOS) in the future. In summary, this study demonstrated that the induction of DDR can also activate expression of transgene(s), via HDACis, in baculovirus-transduced mammalian cells and may be beneficial for the development of BacMam-based gene therapy.

## 4. Materials and Methods

### 4.1. Cells

The *Spodoptera frugiperda* IPBL-Sf21 (Sf21) cell line was cultured in TNM-FH insect medium containing 8% heat-inactivated fetal bovine serum. U-2OS (a human osteogenic sarcoma cell line) cells were grown in McCoy’s medium supplemented with 10% fetal bovine serum.

### 4.2. Construction of Plasmids

The pBac-CMV-αSyn·EGFP was constructed as a vector with fluorescent protein genes to monitor the baculovirus-mediated gene expression in mammalian cells. For this construction, two-step PCR approaches to amplify the α-synuclein gene fused with the EGFP gene (αSyn·EGFP) were used, and the αSyn·EGFP DNA fragment was then cloned into the *Bgl*II- and *Sal*I-digested pBac-CMV-mcs-Lir-EGFP plasmid [36]. The αSyn·EGFP was amplified and rejoined from two PCR products including the αSyn (without a stop codon) fragment and EGFP fragment. The αSyn fragment was PCR amplified from pBac-αSyn-Rhir-SEFP with the forward primer, 5′-GGCAGATCTCCACCATGGATGTATTCATGAAAG-3′ (the *Bgl*II site is underlined) and the reverse primer, 5′-TGCTCACCATACTAGTGGCTTCAGGTTCGTAGTCTTG-3′ (the *Spe*I site is underlined). The EGFP fragment was amplified from pBac-CMV-DsRed-Lir-EGFP with the forward primer, 5′-ACCTGAAGCCACTAGTATGGTGAGCAAGGGCGAG-3′ (the *Spe*I site is underlined), and the reverse primer, 5′-CGCGTCGACGGTATACAGACATG -3′ (the *Sal*I site is underlined). The other EGFP fragment was amplified with the forward primer, 5′-GGCAGATCTCCACCATGGATGTATTCATGAAAG-3′ (the *Bgl*II site is underlined), and the reverse primer, 5′-CGCGTCGACGGTATACAGACATG-3′ (the *Sal*I site is underlined). Then, the PCR product, the αSyn·EGFP fragment, was cloned into the *Bgl*II- and *Sal*I-digested pBac-CMV-mcs-Lir-EGFP plasmid. To construct pBac-CMV-p53-Lir-EGFP as a cancer therapy vector, we used pBac-CMV-mcs-Lir-EGFP as the transfer vector. The p53 gene was amplified from the pEGFP-p53 plasmid (a gift from Yin-Chang Liu, Institute of Molecular Medicine, National Tsing Hua University) with the forward primer, 5′-AAAGATCTCCACCATGGAGGAGCCGCAGTCAG-3′ (the *Bgl*II site is underlined), and the reverse primer, 5′-CCACTAGTTCAGTCTGAGTCAGGCCC-3′ (the *Spe*I site is underlined). Then, the PCR product, the p53 fragment, was cloned into the *Bgl*II- and *Spe*I-digested pBac-CMV-mcs-Lir-EGFP plasmid.

### 4.3. Recombinant Virus Production and Titer Determination

Using Cellfectin (1 μL), the Sf21 cells (2 × 10^5^ cells/well in a 24-well plate) were co-transfected with the linearized viral DNA Bac-N-Blue (0.25 μg; Invitrogen, Carlsbad, CA, USA) and 0.8 μg of one of the transfer vectors, pBac-CMV-αSynEGFP, pBac-CMV-DsRed-Lir-EGFP, or pBac-CMV-p53-Lir-EGFP. The resulting viruses were respectively named vAc-CMV-αSynEGFP, vAc-CMV-DsRed-Lir-EGFP, and vAc-CMV-p53-Lir-EGFP. To isolate vAc-CMV-αSynEGFP, for the Bac-N-Blue viral DNA containing the *lacZ* gene controlled by the ETL promoter, the recombinant virus was identified by X-gal staining according to the manufacturer’s protocol. To isolate vAc-CMV-DsRed-Lir-EGFP and vAc-CMV-p53-Lir-EGFP, they carry the chimeric Liu IRES [36], which contains a tandem repeated TAAG motif that can mediate *EGFP* gene expression in the baculovirus infected Sf21 cells [35], and therefore, both viruses were identified by green fluorescence under a fluorescent microscope. The recombinant viruses were selected and purified by a series of end-point dilutions. Sf21 monolayers were used for virus propagation, and all of the viral stocks were prepared and the titers determined according to the end-point dilution as described previously [46].

### 4.4. Western Blot Analysis

Proteins were separated first by SDS–PAGE on a mini Protein III system (Bio-Rad, Hercules, CA, USA). After SDS–PAGE fractionation, the proteins were electrotransferred onto a polyvinylidene difluoride membrane (Millipore, Bedford, MA, USA). The resulting membrane was blocked with Tris-buffered saline (TTBS; 100 mmol/L Tris (pH 7.4), 100 mmol/L NaCl, and 0.1% Tween 20) containing 5% (*v*/*v*) non-fat dry milk at room temperature for 1 h with gentle shaking. Subsequently, the membrane was incubated with 1:10,000-diluted anti-histone H4 acetylation antibody (GeneTex, Hsinchu, Taiwan) or 1:1000-diluted anti-histone H4 antibody (GeneTex), to detect the epigenetic state of the U-2OS cells. The membrane was incubated with 1:1000-diluted anti-H2AXp antibody (GeneTex) or anti-H2AX antibody (GeneTex) to monitor DNA damage in the U-2OS cells. The membrane was incubated with 1:2500-diluted anti-p53 antibody (GeneTex) to analyze p53 protein expression. All of the antibodies mentioned above were diluted in TBS with 0.5% (*v*/*v*) non-fat, dry milk with shaking at 4 °C overnight. The membranes were also incubated with 1:10,000-diluted anti-actin antibody (Sigma, St Louis, MO, USA) as an internal control to monitor the protein loading for each sample. Unbound antibodies were removed by three washes for 10 min in TTBS buffer at room temperature with shaking. Then, the membrane was incubated with 1:2500-diluted horseradish peroxidase (HRP)-conjugated secondary antibodies (Chemicon, Temecula, CA, USA) for 1 h at room temperature. The HRP on the membrane was detected by an enhanced chemiluminescence kit (Pierce, Rockford, IL, USA) following the protocol provided, and images were captured by the chemiluminescence imaging system (Fusion-SOLO, Newberg, OR, USA). A densitometer scan was performed, and the protein bands were quantified with Image J software 1.48 (W. Rasband, Research Services Branch, NIMH, National Institutes of Health, Bethesda, MD, USA) and normalized to the intensity of actin as the protein loading control for each sample in each experiment.

### 4.5. Transduction of Mammalian Cells

Cells were seeded in 24-well plates at 5 × 10^4^ cells/well. The culture medium was removed and replaced with virus inoculated at a multiplicity of infection of 100 plaque-forming units/cell and centrifuged at 600× *g* for 1 h [47]. Then, the supernatant was removed, and fresh medium containing 1% DMSO, sodium butyrate (NaBt, Sigma), Camptothecin (CTN, Sigma), Teniposide (VM-26, Sigma), or Etoposide (VP-16, Sigma) was added as indicated along with concomitant treatment with the viruses and cultured at 37 °C for 24 h.

### 4.6. EGFP Expression Analysis

For EGFP measurements, cells transduced with vAc-CMV-αSynEGFP were washed with phosphate-buffered saline (PBS) and lysed with 50 μL RIPA buffer (150 mM NaCl, 1% Triton X-100, 0.5% sodium deoxycholate, 0.1% SDS, 50 mM Tris pH 8.0). A 30 μL extract was used for EGFP measurement and a 5 μL extract was used for protein quantity. The green fluorescence intensities were measured using a Cary Eclipse Fluorescence spectrophotometer (Agilent Technologies, Santa Clara, CA, USA). The protein quantities were measured using a BCA protein assay (Thermo Fisher Scientific, Waltham, MA, USA) with BSA as a standard and was used to normalize the green fluorescence intensities. For this purpose, the green fluorescence intensities are normalized with total protein and expressed as fluorescence units per μg protein (FU/μg protein).

### 4.7. Cell Viability Assay

Cell viability measurements were detected by MTT assay. Cells were seeded in a 96-well plate at 1 × 10^4^ cells/well. After 24 h, cells were transduced with mock, vAc-CMV-DsRed-Lir-EGFP or vAc-CMV-p53-Lir-EGFP treatment, and the medium with or without the NaBt or VM-26 was then added and incubated for 24 h. Then, the culture medium was removed and the MTT solution was added to each well. After 1 h of incubation at 37 °C, the medium was removed and 100 μL of dimethyl sulfoxide (DMSO) was then added to each well. Cell viability was determined by measuring the absorbance at 562 nm.

### 4.8. Statistical Analysis

All of the data are presented as the means ± SD. Statistical comparisons of the MTT assay were performed by paired Student’s *t*-tests. *p* < 0.05 indicates a statistically significant difference.
